# New Insights into Bioactive Compounds from the Medicinal Plant *Spathodea campanulata* P. Beauv. and Their Activity against *Helicobacter pylori*

**DOI:** 10.3390/antibiotics9050258

**Published:** 2020-05-15

**Authors:** Corinne Raïssa Ngnameko, Lucia Marchetti, Barbara Zambelli, Antonio Quotadamo, Davide Roncarati, Davide Bertelli, Frederic Nico Njayou, Stella I. Smith, Paul F. Moundipa, Maria Paola Costi, Federica Pellati

**Affiliations:** 1 Department of Life Sciences, University of Modena and Reggio Emilia, Via G. Campi 103, 41125 Modena, Italy; corinnengnameko@yahoo.fr (C.R.N.); lucia.marchetti@unimore.it (L.M.); antonio.quotadamo@unimore.it (A.Q.); davide.bertelli@unimore.it (D.B.); mariapaola.costi@unimore.it (M.P.C.); 2 Department of Biochemistry, Faculty of Science, The University of Yaounde I, P. Box 812 Yaounde, Cameroon; njayou@yahoo.com; 3 Doctorate School in Clinical and Experimental Medicine (CEM), University of Modena and Reggio Emilia, 41125 Modena, Italy; 4 Department of Pharmacy and Biotechnology, Alma Mater Studiorum University of Bologna, Viale Fanin 44, 40127 Bologna, Italy; barbara.zambelli@unibo.it (B.Z.); davide.roncarati@unibo.it (D.R.); 5 Nigerian Institute of Medical Research, PMB 2013, Yaba, Lagos 100001, Nigeria; stellaismith@yahoo.com

**Keywords:** *Spathodea campanulata*, phenolics, UHPLC-MS, *Helicobacter pylori*, urease, adhesins, cytotoxins

## Abstract

The medicinal plant *Spathodea campanulata* P. Beauv. (Bignoniaceae) has been traditionally applied for the prevention and treatment of diseases of the kidney and urinary system, the skin, the gastrointestinal tract, and inflammation in general. The present work shows for the first time how chemical components from this plant inhibit *Helicobacter pylori* growth by urease inhibition and modulation of virulence factors. The crude extract and the main fractions of *S. campanulata* bark were tested on *H. pylori* isolated strains and the active ones were further fractionated. Fractions and sub-fractions of the plant crude extract were characterized by ultra-high-performance liquid chromatographic tandem high resolution-mass spectrometry detection (UHPLC-HRMS). Several phenolics and triterpenoids were identified. Among the sub-fractions obtained, SB2 showed the capacity to inhibit *H. pylori* urease in a heterologous bacterial model. One additional sub-fraction (SE3) was able to simultaneously modulate the expression of two adhesins (HopZ and BabA) and one cytotoxin (CagA). The flavonol kaempferol was identified as the most interesting compound that deserves further investigation as a new hit for its capacity to modulate *H. pylori* virulence factors.

## 1. Introduction

*Helicobacter pylori* (*H. pylori*) is a Gram-negative bacterium that persistently colonizes the stomach of more than half the human population and its prevalence is significantly higher in the developing countries [1,2]. It causes gastric inflammation, gastric mucosa-associated lymphoid tissue lymphoma, and cancer. Due to its role in cancer, it was listed as a class I carcinogen by the World Health Organization (WHO) in 1994 [3].

Several meta-analyses have pointed out that the mass eradication of *H. pylori* would have beneficial effects, such as reduction in gastric cancer incidence, peptic ulcer development, dyspepsia symptoms, and anemia occurrence. Nonetheless, the efficacy of current treatments remains a major concern. The medical therapy for *H. pylori* still relies on a combination of antibiotics and anti-secretory agents, e.g., proton pump inhibitors (PPIs) [4]. However, several studies have described high resistance to antibiotic treatment [5,6,7]. Indeed, in 2017 WHO included *H. pylori* in the list of antibiotic resistant bacterium for which the identification and development of new antimicrobial drugs represent a global priority [8].

To grow in the gastric acid medium *H. pylori* takes advantage of the Ni(II)-dependent urease enzyme, which catalyzes the hydrolysis of urea to produce ammonia and carbamate, the latter subsequently decomposes to ammonia and bicarbonate. The effect of this process is the increase of the medium pH, hence making the environment comfortable for *H. pylori* colonization, despite the harsh acidic conditions of the stomach [9,10]. Urease is therefore a target for the development of alternative and specific antibacterial strategies to overcome *H. pylori* gastric infection.

*H. pylori* uses adhesins to bind and enter to the gastric mucosa. Adhesins are cell-surface proteins that enable bacterial adherence to cells. *H. pylori* major adhesive factors, which belong to the largest outer membrane protein (OMP) family, include blood group antigen-binding adhesion (BabA), sialic acid-binding adhesion (SabA), *H. pylori* outer membrane protein (HopZ), adherence-associated lipoprotein A and B (AlpA-B), *H. pylori* adhesin A (HpaA) and Lewis^x^–LPS. *H. pylori* adhesins are considered bacterial virulence factors and they are involved in several processes during the early and chronic phases of the infection. The most studied virulence factors of *H. pylori* are cytotoxin-associated protein A (CagA) and vacuolating cytotoxin A (VacA) [11,12]. CagA is able to initiate in host cells NF-κB, MAPK, and SHP-2/ERK pathways, producing inflammatory factors and pro-inflammatory cytokines (IL-6, IL-8, INF-γ, TNF-α). These substances may cause extensive infection sites and inflammation, leading to gastritis or gastric cancer [11,12].

The capacity to inhibit the growth of this bacterium has been ascribed to a variety of medicinal plants and natural compounds [13,14]. However, only a few papers have described the mechanisms of action of natural products against *H. pylori* [13,14]. In general, these mechanisms include urease activity inhibition, anti-adhesion activity, DNA damage, protein synthesis inhibition, and oxidative stress [15,16,17]. 

*Spathodea campanulata* P. Beauv. (Bignoniaceae) is a medicinal plant traditionally used in Africa for the prevention and treatment of diseases of the kidney and urinary systems, the skin, the gastrointestinal tract, and inflammation in general [18,19]. Extracts of this plant have been found to be active against proliferative diseases, involving cancer cells and bacteria [20]. More recently, anti-*H. pylori*, anti-adhesive, and growth inhibitory activities of a methylene chloride (DCM)/methanol (MeOH) extract from the bark of the plant have been described [21]. Data about the chemical characterization of *S. campanulata* are limited. Nevertheless, previous studies undertaken on the stem bark and leaves have shown the presence of phenolic acids, flavonoids, triterpenoids, iridoids, and sterols [22,23,24,25,26,27].

Consequently, this is the first chemical characterization study of the main compounds present in the extracts, fractions and sub-fractions of *S. campanulata* bark using UHPLC-HRMS, which were also assessed for their anti-*H. pylori*, anti-urease, and anti-adhesins effects.

## 2. Materials and Methods

### 2.1. Chemicals and Solvents

Analytical grade solvents, HPLC grade solvents and ammonium formate were from Sigma–Aldrich (Milan, Italy). Ultrapure water (H_2_O) was obtained from a Millipore water purification system (Merck Life Science, Milan, Italy). Pre-coated aluminum Kieselgel 60 F_254_ plates (layer thickness 0.2 mm, Merck, Darmstadt, Germany) were used for analytical thin-layer chromatography (TLC).

### 2.2. Plant Material and Preparation of the Crude Extract

The bark from *S. campanulata* was collected in July 2018 in Foumbot (West Region, Cameroon). A sample of the bark was deposited at the HNC-Cameroon National Herbarium, with the voucher number 50085/HNC. The bark used in this study was harvested from at least three different trees, in order to have a representative sample. The plant material was washed with H_2_O and dried at room temperature for several weeks. The dried plant material was then powdered using a grinder. The obtained powder was kept at 4 °C until the preparation of the extracts. A portion of 500 g of powdered plant material was soaked in 2 L of solvent solution composed by DCM/MeOH (1:1, *v/v*) for 48 h and paper filtered. The filtrate was dried using a rotary evaporator to obtain the crude extract.

### 2.3. Fractionation Procedure

The crude extract was purified on silica gel by preparative flash column chromatography using in sequence *n*-hexane (Hex), ethyl acetate (EtOAc), and MeOH. Eight stages of polarity were used: Hex (fraction A), Hex/EtOAc 25% (fraction B), Hex/EtOAc 50% (fraction C), Hex/EtOAc 75% (fraction D), EtOAc (fraction E), and MeOH (fraction F). The fractions were further partitioned using a silica gel column chromatography with an Isolera apparatus (Biotage, Charlotte, NC, USA) with different solvents, according to the result of TLC. In this way, different sub-fractions were collected: SA1 (90% Cyhex/EtOAc) from fraction A, SB1 and SB2 (70% Cyhex/EtOAc and 90% Cyhex/EtOAc, respectively) from fraction B, SC1 and SC2 (95% DCM/MeOH) from fraction C, SD1 and SD2 (20% Cyhex/EtOAc) from fraction D, SE1 and SE3 (50% MeOH/H_2_O and 70% MeOH/H_2_O, respectively) from fraction E, and SF (80% Cyhex/EtOAc) from fraction F. The solvent was removed from the collected fractions and sub-fractions by using a rotary evaporator.

### 2.4. Determination of Total Polyphenols and Total Flavonoids

For the determination of total polyphenols and total flavonoids, the crude extract and its fractions were dissolved in MeOH at a concentration of 100 µg/mL. The content of total polyphenols was analyzed according to the Folin–Ciocalteu’s method, as previously described [28]. Briefly, 50 µL of sample was added to a solution composed of 2.4 mL of distilled water, 200 µL of Folin–Ciocalteu’s reagent (2 N), and 500 µL of Na_2_CO_3_ 20%. The reaction mixture was incubated at 25 °C in dark for 1 h, and the absorbance was read at 765 nm. The results were compared to a gallic acid calibration curve, and the total phenolic content was expressed as milligrams of gallic acid equivalents (GAE) per gram of extract.

The total flavonoid content was analyzed according to the aluminum chloride method [28]. Briefly, 50 µL of sample was mixed with 0.95 mL of MeOH and 1.0 mL of AlCl_3_ (2%). The reaction mixture was incubated at 25 °C for 30 min, and the absorbance was read at 420 nm. The results were compared to quercetin calibration curve and the total flavonoids content was expressed as milligrams of quercetin equivalents (QE) per gram of extract.

### 2.5. UHPLC-HRMS Analysis

For UHPLC-HRMS analysis, 2 mg of each fraction and sub-fraction obtained from the crude plant extract were dissolved in 1.0 mL of acetonitrile (ACN), filtered using a PTFE filter, and vortex mixed for 20 s, spun at 5000 g for 5 min. Then, 5 µL of this solution were injected in duplicate, for the analysis. A Thermo Scientific Dionex Ultimate 3000 UHPLC system was used, equipped with an autosampler, a quaternary pump and a thermostated column compartment controlled by the Chromeleon 7.2 Software (Thermo Scientific, Waltham, MA, USA). The UHPLC system was coupled to a high-resolution Q Exactive mass spectrometer (Thermo Scientific, Bremen, Germany). The mass spectrometer was calibrated before the analyses. Nitrogen (N_2_) (purity > 99.999%), obtained from a Zefiro zero 60 LC-MS nitrogen generator (CINEL, Vigonza, Italy), was employed both as the source gas and the collision gas. The chromatographic system was hyphenated to the MS with an electrospray ionization (ESI) source, which was operated both in the positive and in the negative ion modes. The capillary temperature was set at 270 °C and the following N_2_ flows (arbitrary units) were used: sheat gas 40, auxiliary gas 30, and sweep gas 3. The capillary voltage was set at −3.5 and +3 kV. The chromatographic separation was performed on an Ascentis Express C_18_ (10 cm × 2.1 mm i.d., 2.7 µm) (Supelco, Bellefonte, PA, USA). The column was kept at 25 °C. The mobile phase was composed of 80% H_2_O + 0.1% formic acid (FA) (A) and 20% ACN (B) at 0.3 mL/min. The following gradient was used: from 0.1 to 10.1 min from 20% to 98% B, which was kept constant for 8.8 min, before the reconditioning step, for a total analysis time of 25 min. 

MS and MS^2^ spectra were recorded from 100 to 1000 *m*/*z*, at a resolution of 70,000 and 17,500, respectively. The three most intense ions were selected for MS^2^ N_2_-promoted collision-induced dissociation (CID) (stepped CE = 20, 28 eV). A precursor active exclusion of 6 s was set. In parallel, an all ion fragmentation experiment (CE = 28 eV) was scheduled for post-hoc analysis. The mass calibration was performed weekly, both in the negative and in the positive ion modes, to ensure a mass accuracy ≤ 2 ppm. XCalibur 2.9 (Thermo Scientific, Bremen, Germany) and FreeStyle 1.3 software (Thermo Scientific, San José, CA, USA) were used for UHPLC control and MS data processing, respectively.

### 2.6. Antimicrobial Disk Diffusion Test

*H. pylori* strain G27 was obtained from the University of Bologna, Italy. *H. pylori* cells were recovered from glycerol stocks on Brucella broth agar plates, containing 5% fetal calf serum (FCS), added with Dent’s antibiotic supplement in an atmosphere of 9% CO_2_/91% air, maintained at 37 °C, and 95% humidity in water-jacketed incubator (Thermo Fisher Scientific, Waltham, MA, USA). All reagents were purchased from Oxoid, United Kingdom. The assay was carried out following the method described by Balouiri et al. [29]. *H. pylori* cells were collected from Brucella broth agar plates, suspended in 500 µL of liquid Brucella broth with 5% Dent’s antibiotic supplement and subsequently the cell density (OD600) of bacterial suspension was determined. Then, *H. pylori* cells were diluted in melted Brucella broth Soft Agar medium (Brucella broth agar plates containing 0.5% agar) to obtain a final OD600 of 0.07 and 6.5 mL of this suspension was poured into a sterile standard Brucella broth agar plate. After that, 5 mm sterile paper disks were deposited into the agar. A total of 4 µL of the crude extract, fractions, and sub-fractions (10 mg/mL), prepared using DMSO/H_2_O (25% v/v), were dropped into the disk. The DMSO/H_2_O solution was used as the negative control. Kanamycin (10 µg) was used as the reference antibiotic. The plates were incubated under microaerophilic conditions at 37 °C for 72 h. The products that showed a diameter of inhibition ≥ 8 mm were considered to be active [29]. The active products were serially diluted using sterile DMSO/H_2_O (25% v/v) to obtain three decreasing concentrations, which were re-tested in triplicate and the results expressed as a mean diameter of inhibition zone. The minimum active quantity (MAQ) for each extract was determined as the minimum quantity that produced an inhibition zone ≤ 8 mm.

### 2.7. In-Cell Urease Activity Test

*Escherichia coli* TOP10 cells harboring pGEM-ureOP plasmid were pre-cultured at 37 °C in 1 mL of lysogeny broth (LB), containing 100 μg/mL of ampicillin (Ap). After 16 h, 40 μL of pre-culture were used to inoculate 2 mL of LB medium containing 100 μg/mL of Ap and 10 μg/L of cresol red pH indicator. When OD600 reached 0.7–0.9, 90 μL of each culture was inoculated in 96-well plates with 80 mM urea, with and without 10 µL of different concentrations of either the fractions or the sub-fractions of the plant extract or DMSO. Immediately before running the experiment, 2.5 µL of 10 mM NiSO_4_ was added to each culture to a final concentration of 250 µM. In each well, 30 μL of mineral oil was added to prevent the medium evaporation during the culture. The color change of cresol red indicator was monitored over time spectrophotometrically in a multi-plate reader, measuring the absorption at 430 and 580 nm.

### 2.8. Western Blot Analysis

Three days old cultures of the *H. pylori* G27 strain were harvested into Brucella broth medium supplemented with 10% FBS to 0.8 OD600. Then, 100 µL of the mixture was added to each well in a 96-well plate. A total of 100 µL of the plant extract at the three concentrations was used. The plates were incubated at 37 °C under microaerophilic conditions for 4 h. After incubation, 200 µL of the mixture was transferred into a microcentrifuge tube and centrifuged at 14,000 rpm for 5 min at 4 °C. The supernatant was discarded, and the pellet was washed with phosphate buffer saline (PBS). Protein in Laemmli loading buffer was separated by 12% sodium dodecyl sulfate poly-acrylamide gel electrophoresis (SDS-PAGE) and electro-transferred into a polyvinylidene difluoride paper (PVDF) blotting membrane (GE Healthcare, Germany). The membranes were blocked with 5% w/v non-fat milk in phosphate buffered saline Tween-20, incubated overnight at 4 °C with primary antibodies (BabA, CagA, HopZ, and HP1043 as the loading control), rinsed, and then incubated for 1 h at 25 °C with horseradish peroxidase-conjugated secondary antibodies. The blots were then developed by using a chemiluminescent method (Sigma, Germany). The densitometric analysis of the protein bands was performed using the ImageJ Software (NIH, USA).

### 2.9. Statistical Analysis

Data analysis was carried out using the GraphPad Prism 5.0 software. For the diameter of inhibition, the results were reported as mean ± standard deviation (SD). For the IC_50_ of urease and adhesion, the results were expressed as mean ± SD of three independent experiments. Regarding protein expression, data were presented as mean ± SD. Comparisons were made between untreated group and DMSO control groups.

## 3. Results and Discussion

### 3.1. Chemical Composition of S. campanulata Fractions and Sub-Fractions

To identify new anti-*H. pylori* growth inhibitors, we first tested fractions and sub-fractions of bark of *S. campanulata* against the bacterium and then we focused on the urease enzyme and adhesin proteins involved in adhesion to gastric mucosa.

After the extraction of the plant material, its fractionation and further sub-fractionation were performed by means of preparative flash column chromatography. Six fractions were obtained, which were further sub-fractioned using silica gel column chromatography to provide 10 sub-fractions. The content of total polyphenols and total flavonoids in the crude plant extract and in the six fractions is shown in Table 1.

MS and MS/MS data of all the identified compounds are shown in Table S1. The chemical structure of the 11 characterized compounds, which was confirmed by comparison with existing structures [22,23,24,25,26,27,30], is shown in Table 2. Compound 5,7-dihydroxy-4-metilcoumarin was detected in this plant for the first time, through the mass spectral database mzCloud.

### 3.2. Inhibitory Activity of S. campanulata Fractions and Sub-Fractions against H. pylori

The inhibitory activity on *H. pylori* of all the fractions and sub-fractions is shown in Table 3. The minimum active quantity (MAQ) values ranged from 0.1 mg to 10 mg/mL. The lowest MAQ value of 0.1 mg/mL was obtained with fractions B, C, and E and sub-fractions SA1 and SB1 on *H. pylori*. 

**Figure 1:**
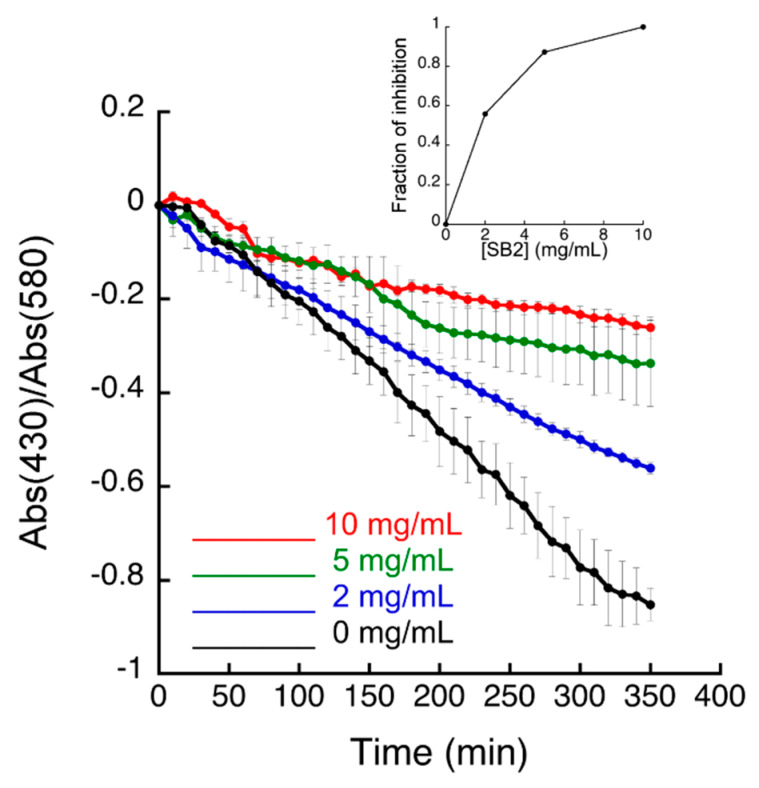
Urease activity of recombinant *Escherichia coli* cells in the presence of 250 μM Ni(II) and of 80 mM urea, measured as a change of pH detected by the cresol red indicator. Increasing concentrations of the sub-fraction SB2 were added to the *E. coli* culture before performing the colorimetric assay. Data are shown as mean ± SD of the triplicates. The insert represents the dose-response plot for the SB2 sub-fraction. Sub-fraction SB2 (90% Cyhex/EtOAc) was obtained from fraction B.

The measurement of the cellular urease activity was performed in a heterologous *E. coli* bacterial model overexpressing the enzyme (Figure 1, Figures S1–S3), as previously described [31]. 

The results indicated that sub-fractions SD2, SE1 and SE3 produced minor urease inhibition (Figures S1–S3), while the sub-fraction SB2 inhibited urease in living bacteria with an IC_50_ value of 3.3 mg/mL (Figure 1). All the other fractions showed either low solubility or limited activity.

### 3.3. Effect of S. campanulata Fractions and Sub-Factions on H. pylori Virulence Factors

**Figure 2:**
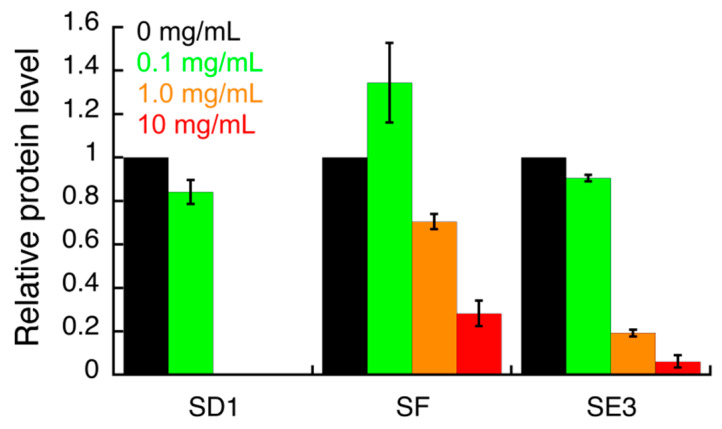
Densitometric analysis of the Western blot images (shown in the Supplementary Materials) indicating the effects of *S. campanulata* on *H. pylori* G27 HopZ adhesin expression. *H. pylori* cells were treated with different concentrations of *S. campanulata* sub-fractions for 4 h and protein level of HopZ adhesin was determined by Western blot, using a specific anti-HopZ antibody. Values are expressed as means ± SD of two independent experiments Sub-fraction SD1 (20% Cyhex/EtOAc), sub-fraction SE3 (70% MeOH/H_2_O), and sub-fraction SF (80% Cyhex/EtOAc) were from fractions D, E, and F, respectively.

**Figure 3:**
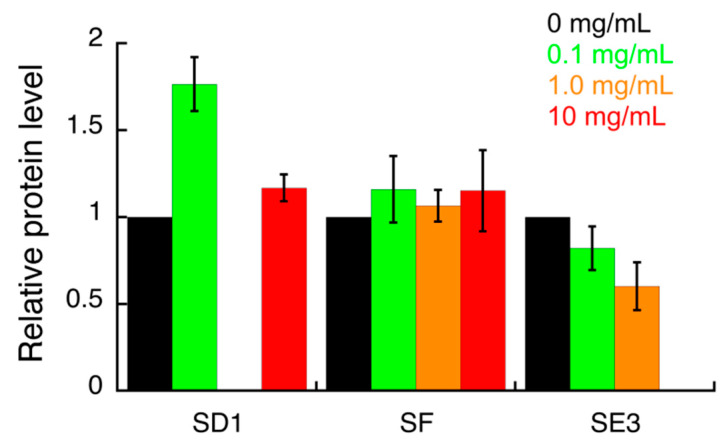
Densitometric analysis of the Western blot images (shown in the Supplementary Materials) indicating the effects of *S. campanulata* on *H. pylori* G27 BabA adhesin expression. *H. pylori* cells were treated with different concentrations of *S. campanulata* sub-fractions for 4 h and protein level of BabA adhesin was determined by Western blot, using a specific anti-BabA antibody. Values are expressed as means ± SD of two independent experiments. Sub-fraction SD1 (20% Cyhex/EtOAc), sub-fraction SE3 (70% MeOH/H_2_O), and sub-fraction SF (80% Cyhex/EtOAc) were from fractions D, E, and F, respectively.

**Figure 4:**
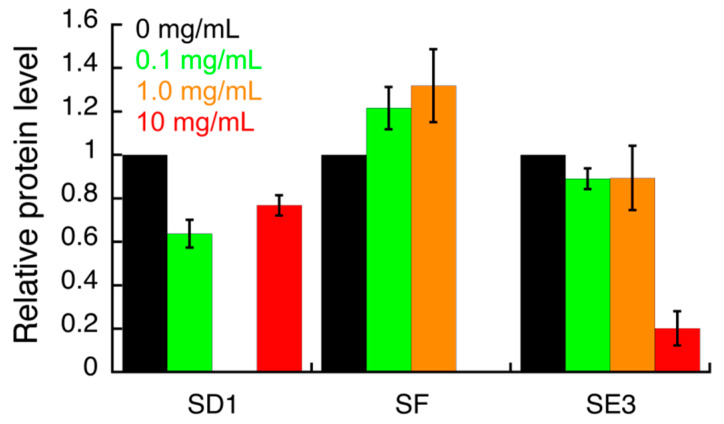
Densitometric analysis of the Western blot images (shown in the Supplementary Materials) indicating the effects of *S. campanulata* on *H. pylori* G27 CagA expression. *H. pylori* cells were treated with different concentrations of *S. campanulata* sub-fractions for 4 h and protein level of the virulence factor CagA was determined by Western blot, using a specific anti-CagA antibody. Values are expressed as means ± SD of two independent experiments. Sub-fraction SD1 (20% Cyhex/EtOAc), sub-fraction SE3 (70% MeOH/H_2_O), and sub-fraction SF (80% Cyhex/EtOAc) were from fractions D, E, and F, respectively.

The effect of all *S. campanulata* fractions and sub-fractions on the expression of specific *H. pylori* virulence factors, including the two adhesins BabA and HopZ, and the cytotoxin-associated gene A (CagA), was investigated by Western blot. The samples that showed a significant effect in this preliminary assay were further investigated (Figure S4; Figure 2, Figure 3 and Figure 4). HP1043 staining was used as the loading control. Densitometric analysis of blot was performed and quantification data are reported in the bar chart. Values are expressed as means ± SD of two independent experiments. Specifically, sub-fractions SD1, SE3, and SF were able to inhibit HopZ expression (Figure 2). Sub-fractions SD1 and SE3 were found to slightly inhibit the expression of BabA (Figure 3). As for CagA expression, only sub-fraction SE3 inhibited the expression of this cytotoxin (Figure 4). 

The compounds identified in this work by UHPLC-HRMS may be involved in the observed biological activity either by inhibiting urease activity or by modulating the expression of the virulence factors mentioned above. The plant sub-fraction SE3, able to simultaneously modulate the expression of two adhesins (HopZ and BabA) and one cytotoxin (CagA), was obtained from fraction E, which was the richest one in total polyphenols and total flavonoids (Table 1); the flavonol kaempferol was identified in sub-fraction SE3 by UHPLC-HRMS analysis. As for the other two sub-fractions SD1 and SF, their chemical composition was not disclosed by UHPLC-HRMS analysis. 

Phenolics have general antimicrobial activity and some of them are also active against *H. pylori* [13,32]. Several phytochemicals may act by inhibiting enteric microbial growth, inducing cellular membrane perturbations, interfering with certain microbial metabolic processes and modulating signal transduction or gene expression pathways [32]. Notably, it has been recently observed that the flavonoid kaempferol, a widely diffused natural flavonol, has an anti-inflammatory effect on *H. pylori*-induced inflammation by suppressing the translocation of CagA and VacA proteins and leading to the downregulation of pro-inflammatory cytokines [33]. Recently, kaempferol has been found to inhibit the *in vitro* DNA binding activity of the homeostatic stress regulator (HsrA), a protein which plays as a global homeostatic regulator, synchronizing metabolic functions and virulence with availability of nutrients and cell division, mediating also the response to oxidative stress. HsrA represents a novel and effective therapeutic target in *H. pylori* infection [34]. In the present work, the sub-fraction SE3 from *S. campanulata*, which contains kaempferol, was found to simultaneously modulate the expression of the two *H. pylori* adhesins HopZ and BabA and the cytotoxin CagA; in addition, this sub-fraction showed a mild urease inhibition. Regarding the effect of natural compounds on the expression of HopZ, to the best of our knowledge no information is available in the literature. N-phenylpropenoyl-l-amino acids (NPAs) has been identified as polyphenol/amino acid conjugates in the seeds of theobroma cacao as well as in a variety of herbal drugs; they have been described for their inhibitors of *H. pylori* BabA outer membrane protein [35]. As for CagA, in addition to kaempferol, binding interactions between this virulence factor and natural compounds have been investigated by molecular docking and four hit compounds have been identified, including one phytosterol, one chalcone, and two carotenoids [36]. Due to the reduced number of natural compounds able either to inhibit urease enzymatic activity or to modulate the expression of *H. pylori* virulence factors, the research in this field deserves a great attention in the medicinal chemistry field in order to develop the identified hit kaempferol, alone or in combination with known drugs. This outcome is in agreement with a recent study [34], where kaempferol has exhibited a potent bactericidal activity against *H. pylori*, with an MBC value from 28 to 56 µM, the latter in resistant strains. However, the wide group of intracellular targets and the high number of natural compounds potentially effective as therapeutic agents against *H. pylori* present in this plant extract require further evidence to better understand the role of these substances using in vivo models, as previously described in the literature for kampferol isolated from *Polygonum tinctorium* Lour. [37].

## 4. Conclusions

It can be assumed from this study that natural compounds present in *S. campanulata* bark extract could be effective as potential therapeutic agents against *H. pylori*, by inhibiting urease activity and some virulence factors and by modulating the expression of the adhesins BabA and HopZ and the cytotoxin CagA. These results suggest that enriched extracts from this plant could be useful for the treatment of *H. pylori* mediated gastric diseases and they could be used synergistically with antibiotics in conventional therapy. The flavonol kaempferol, identified by UHPLC-HRMS, has to be further assessed as a new hit for its potential therapeutic activity against *H. pylori* infection.

## Figures and Tables

**Table 1:** Total polyphenols and total flavonoids content in *Spathodea campanulata* crude extract and fractions (A–F).

**Total Polyphenols (mg GAE/g) ^a^**						
Crude extract	A	B	C	D	E	F
51.4 ^b^	46.4 ± 0.5	43.0 ± 0.1	53.0 ^b^	48.3 ^b^	68.8 ± 0.3	56.3 ± 1.1
**Total Flavonoids (mg QE/g) ^a^**						
Crude extract	A	B	C	D	E	F
5.2 ± 0.2	2.8 ± 0.1	1.8 ^b^	1.9 ^b^	2.0 ^b^	7.1 ^b^	1.9 ± 0.1

^a^ Data of total polyphenols are expressed as mg of gallic acid equivalents (GAE)/g; data of total flavonoids as mg of quercetin equivalents (QE)/g (± SD). ^b^ SD < 0.05.

**Table 2:** Chemical structures of the identified compounds and their distribution in the active fractions and sub-fractions of *Spathodea campanulata*.

Compound	Structure	Fraction/Sub-Fraction							
		C	E	SA1	SB1	SC2	SD2	SE1	SE3
**4-Hydroxy Benzoic Acid**	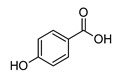								
**Methyl-4-Hydroxybenzoate**	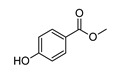								
**5,7-Dihydroxy-4-Metilcoumarin**	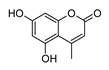								
**Quercetin**	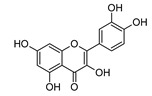								
**Kaempferol**	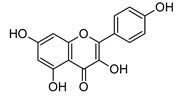								
**Kaempferol 3-*O*-Glucoside**	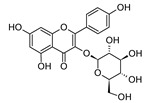								
**Spathodol**	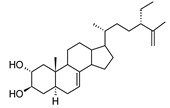								
**Spathodic Acid**	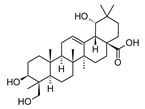								
**Tomentosolic Acid**	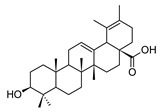								
**Ursolic Acid**	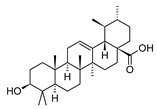								
**Corosolic Acid**	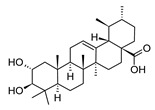								

**Table 3:** Mean diameter of inhibition (mm ± SD) and minimum active quantity (MAQ) values (mg/mL) of *Spathodea campanulata* crude plant extract, active fractions and sub-fractions against *Helicobacter pylori*.

Sample.	Concentration			
	0.1 mg/mL	1.0 mg/mL	10.0 mg/mL	MAQ (mg/mL)
Crude extract	ND	8.6 ± 0.8	11.6 ± 0.7	1.0
B	11.3 ± 1.2	11.7 ± 2.1	12.1 ± 1.0	0.1
C	10.0	12.0	16.3 ± 2.5	0.1
E	10.0	11.7 ± 0.6	15.8 ± 0.3	0.1
SA1	9.8 ± 0.4	10.0 ± 1.0	10.7 ± 1.2	0.1
SB1	9.3 ± 0.6	9.7 ± 0.6	10.7 ± 0.6	0.1
SB2	ND	8.7 ± 2.3	11.3 ± 2.5	1.0
SC2	ND	8.0	10.0	1.0
SD1	ND	ND	10.3 ± 0.6	10.0
SE3	ND	ND	13.0 ± 1.0	10.0
SF	ND	9.3 ± 1.2	10.7 ± 0.6	1.0
Kanamycin (2.5 mg/mL)	35.7 ± 0.6	-	-	-

## Supplementary Materials

The following files are available online at https://www.mdpi.com/2079-6382/9/5/258/s1, Figure S1: Urease activity of recombinant *E. coli* cells in the presence of 250 μM Ni(II) and of 80 mM urea, measured as a change of pH detected by the cresol red indicator. Increasing concentrations of the sub-fraction SD2 were added to the *E. coli* culture before performing the colorimetric assay. Data are shown as mean ± SD of the triplicates. Sub-fraction SD2 (20% Cyhex/EtOAc) was obtained from fraction D. Figure S2: Urease activity of recombinant *E. coli* cells in the presence of 250 μM Ni(II) and of 80 mM urea, measured as a change of pH detected by the cresol red indicator. Increasing concentrations of the sub-fraction SE1 were added to the *E. coli* culture before performing the colorimetric assay. Data are shown as mean ± SD of the triplicates. Sub-fraction SE1 (50% MeOH/H_2_O) was obtained from fraction E. Figure S3: Urease activity of recombinant *E. coli* cells in the presence of 250 μM Ni(II) and of 80 mM urea, measured as a change of pH detected by the cresol red indicator. Increasing concentrations of the sub-fraction SE3 were added to the *E. coli* culture before performing the colorimetric assay. Data are shown as mean ± SD of the triplicates. Sub-fraction SE3 (70% MeOH/H_2_O) was obtained from fraction E. Figure S4: Western blot analysis of *H. pylori* cells, treated with various concentrations of sub-fractions, to quantify the expression levels of HopZ, BabA and CagA proteins. DMSO was used as the control treatment (C), while HP1043 staining was used as the loading control. Sub-fraction SD1 (20% Cyhex/EtOAc), sub-fraction SE3 (70% MeOH/H_2_O) and sub-fraction SF (80% Cyhex/EtOAc) were from fractions D, E and F, respectively. Table S1: Compounds identified in the active fractions and sub-fractions from *Spathodea campanulata* by UHPLC-HRMS.
