# Supplementary material for: New Insights into Bioactive Compounds from the Medicinal Plant Spathodea campanulata P. Beauv. and Their Activity against Helicobacter pylori
Source: Antibiotics (Basel). 2020 May 15;9(5):258. doi: 10.3390/antibiotics9050258 (PMC7277392; doi:10.3390/antibiotics9050258)
Supplement: Supplementary file 1 [file antibiotics-09-00258-s001.zip › Supplementary revised/Supplementary figure S4-DAV.docx]

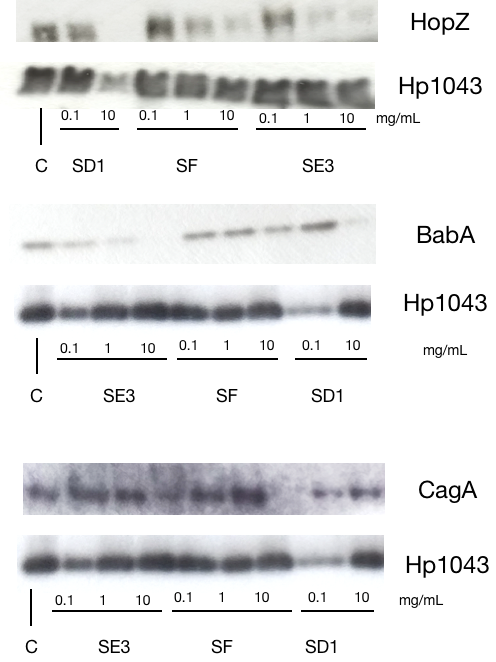


**Figure S4.** Western blot analysis of *H. pylori* cells, treated with various concentrations of sub-fractions, to quantify the expression levels of HopZ, BabA and CagA proteins. DMSO was used as the control treatment (C), while HP1043 staining was used as the loading control. Sub-fraction SD1 (20% Cyhex/EtOAc), sub-fraction SE3 (70% MeOH/H_2_O) and sub-fraction SF (80% Cyhex/EtOAc) were from fractions D, E and F, respectively.
