# Supplementary material for: New Insights into Bioactive Compounds from the Medicinal Plant Spathodea campanulata P. Beauv. and Their Activity against Helicobacter pylori
Source: Antibiotics (Basel). 2020 May 15;9(5):258. doi: 10.3390/antibiotics9050258 (PMC7277392; doi:10.3390/antibiotics9050258)
Supplement: Supplementary file 1 [file antibiotics-09-00258-s001.zip › Supplementary revised/Supplementary figures S1-3.docx]

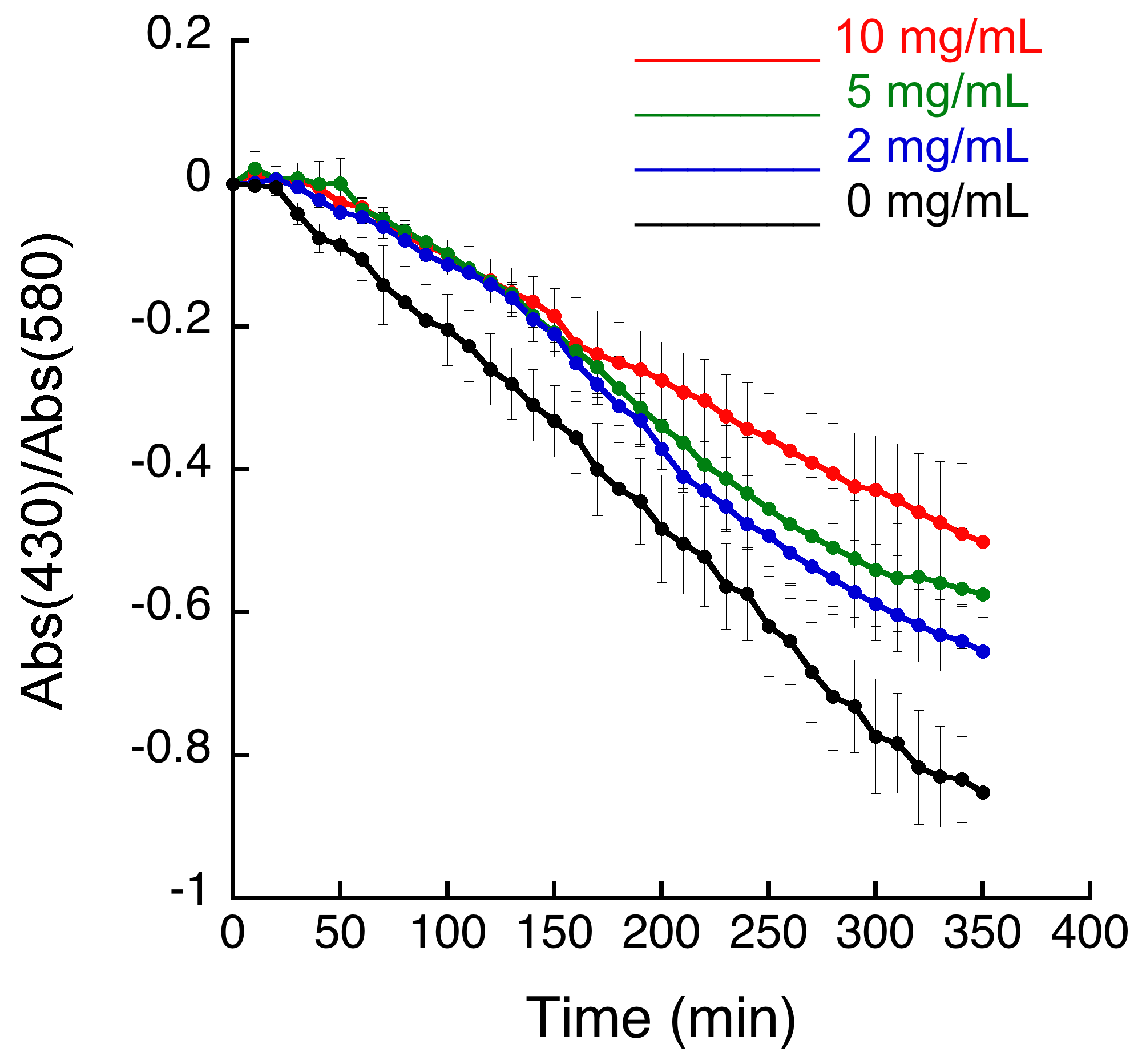


**Figure S1**. Urease activity of recombinant *E. coli* cells in the presence of 250 μM Ni(II) and of 80 mM urea, measured as a change of pH detected by the cresol red indicator. Increasing concentrations of the sub-fraction SD2 were added to the *E. coli* culture before performing the colorimetric assay. Data are shown as mean ± SD of the triplicates. Sub-fraction SD2 (20% Cyhex/EtOAc) was obtained from fraction D.


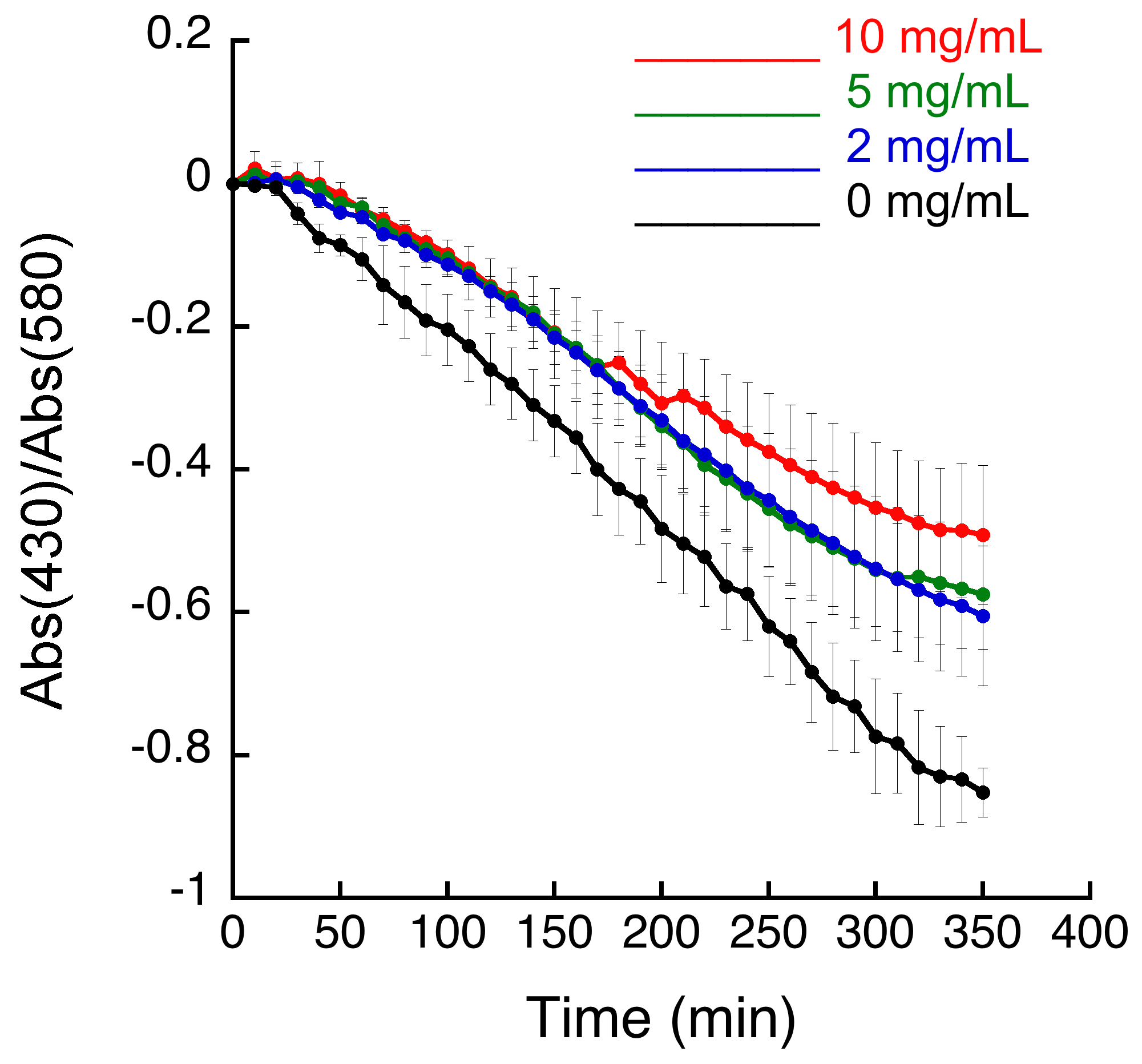


**Figure S2**. Urease activity of recombinant *E. coli* cells in the presence of 250 μM Ni(II) and of 80 mM urea, measured as a change of pH detected by the cresol red indicator. Increasing concentrations of the sub-fraction SE1 were added to the *E. coli* culture before performing the colorimetric assay. Data are shown as mean ± SD of the triplicates. Sub-fraction SE1 (50% MeOH/H_2_O) was obtained from fraction E.


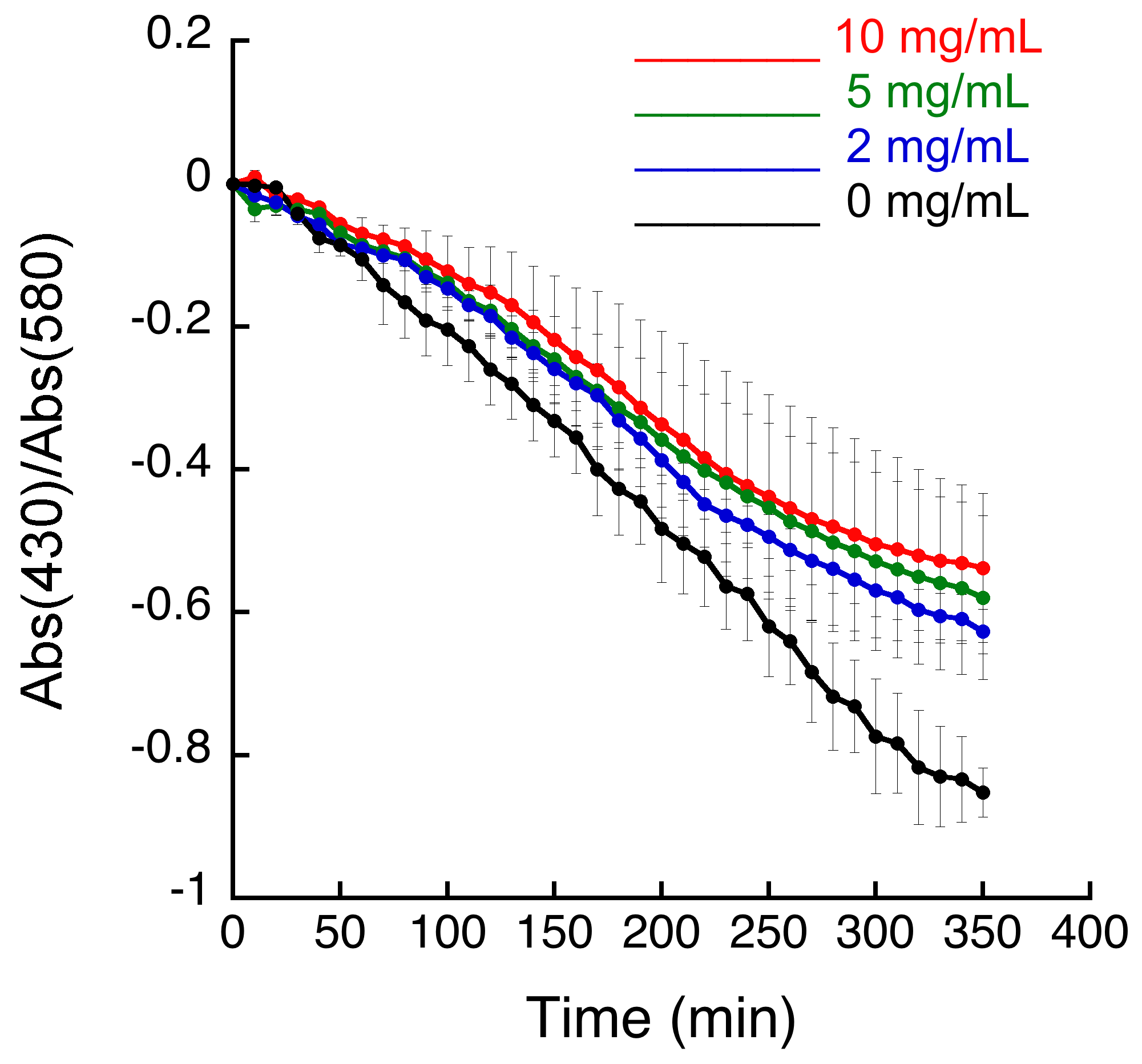


**Figure S3**. Urease activity of recombinant *E. coli* cells in the presence of 250 μM Ni(II) and of 80 mM urea, measured as a change of pH detected by the cresol red indicator. Increasing concentrations of the sub-fraction SE3 were added to the *E. coli* culture before performing the colorimetric assay. Data are shown as mean ± SD of the triplicates. Sub-fraction SE3 (70% MeOH/H_2_O) was obtained from fraction E.
