# Supplementary material for: New Insights into Bioactive Compounds from the Medicinal Plant Spathodea campanulata P. Beauv. and Their Activity against Helicobacter pylori
Source: Antibiotics (Basel). 2020 May 15;9(5):258. doi: 10.3390/antibiotics9050258 (PMC7277392; doi:10.3390/antibiotics9050258)
Supplement: Supplementary file 1 [file antibiotics-09-00258-s001.zip › Supplementary revised/Supplementary table S1.docx]

| **Table S1**  **Compounds identified in the active fractions and sub-fractions from *Spathodea campanulata* by UHPLC-HRMS** | | | | | | |
| --- | --- | --- | --- | --- | --- | --- |
| **Fraction** | **Ion**  **mode** | **Precursor ion** | **Fragments** | **Molecular**  **Formula** | **Identification** | **Literature** |
| C | [M-H]^-^ | 191.0342 | 176.0106,148.0155,104.0255 | C_10_H_8_O_4_ | Tentative identification:  5,7-dihydroxy-4-metilcoumarin |  |
| C | [M-H]^-^ | 137.0235 | 105.4586,93.0333 | C_7_H_6_O_3_ | 4-hydroxy-benzoic acid | Pianaro et al. 2007 |
| C | [M-H]- | 301.0354 | 273.0408,245.0457,178.9977,151.0026 | C_15_H_10_O_7_ | Quercetin | Boniface et al. 2019 |
| C | [M-H]^-^ | 285.0405 | 151.0025,133.0283 | C_15_H_10_O_6_ | Kaempferol | Lifongo et al. 2014 |
| C | [M+H]^+^ | 139.0391 | 95.0497,91.0548,67.0550 | C_7_H_6_O_3_ | 4-hydroxy-benzoic acid | Pianaro et al. 2007 |
| C | [M+H]^+^ | 193.0498 | 178.0261,165.0549,150.0312,  133.0285,122.0365 | C_10_H_8_O_4_ | Tentative identification:  5,7-dihydroxy-4-metilcoumarin |  |
| C | [M+H]^+^ | 147.0442 | 103.0547,91.0548,77.0392,65.0394 | C_9_H_6_O_2_ | Coumarin | Boniface et al. 2015 |
| C | [M+H]^+^ | 287.0552 | 269.0440,153.0183,137.0232 | C_15_H_10_O_6_ | Kaempferol | Lifongo et al. 2014 |
| C | [M+H]^+^ | 111.0445 | 93.0703,65.0394,55,0551 | C_6_H_6_O_2_ | Catechol | Boniface et al. 2015 |
| SC2 | [M-H]^-^ | 191.0342 | 176.0106,148.0155,104.0255 | C_10_H_8_O_4_ | Tentative identification:  5,7-dihydroxy-4-metilcoumarin |  |
| SC2 | [M-H]^-^ | 285.0405 | 151.0025,133.0283 | C_15_H_10_O_6_ | Kaempferol | Lifongo et al. 2014 |
| SC2 | [M+H]^+^ | 139.0391 | 95.0497,91.0548,67.0550 | C_7_H_6_O_3_ | 4-hydroxy-benzoic acid | Pianaro et al. 2007 |
| SC2 | [M+H]^+^ | 287.0552 | 269.0440,153.0183,137.0232 | C_15_H_10_O_6_ | Kaempferol | Lifongo et al. 2014 |
| E | [M+H]^+^ | 447.0933 | 285.0402,151.0027 | C_21_H_20_O_11_ | Kaempferol 3-glucoside | Nazif 2007 |
| E | [M-H]^-^ | 285.0405 | 229.2277,151.0029,133.0284 | C_15_H_10_O_6_ | Kaempferol | Lifongo et al. 2014 |
| E | [M-H]^-^ | 455.3531 | 411.3633 | C_30_H_48_O_3_ | Ursolic acid | Lifongo et al. 2014  Amusan et al. 1996  Heim et al. 2012 |
| E | [M+H]^+^ | 153.0546 | 123.0441,95.0132,79.0184 | C_8_H_8_O_3_ | Methyl 4-hydroxybenzoate | Pianaro et al. 2007 |
| E | [M+H]^+^ | 193.0498 | 178.0258,150.0311,133.0283,137.0595 | C_10_H_8_O_4_ | Tentative identification:  5,7-dihydroxy-4-metilcoumarin |  |
| E | [M+H]^+^ | 449.1078 | 287.0551 | C_21_H_20_O_11_ | Kaempferol 3-glucoside | Nazif 2007 |
| E | [M+H]^+^ | 455.3516 | 437.3415,409.3470,391.3368,219.1742 | C_30_H_46_O_3_ | Tomentosolic acid | Amusan et al. 1996  Heim et al. 2012 |
| E | [M+H]^+^ | 457.3313 | 439.3628,411.3253,393.3140,339.2325,  175.1483 | C_30_H_48_O_3_ | Ursolic acid | Lifongo et al. 2014  Amusan et al. 1996  Heim et al. 2012 |
| E | [M+H]^+^ | 429.3725 | 412.3649,346.2809,317.2466,303.2307, 289.21592,177.0911,151.0753,137.0596 | C_29_H_48_O_2_ | Tentative identification:  Spathodol | Heim et al. 2012  Ngouela et al. 1991 |
| E | [M+H]^+^ | 489.3585 |  | C_30_H_48_O_5_ | Tentative identification:  Spathodic acid | Heim et al. 2012 |
| SA1 | [M-H]^-^ | 455.3531 | 411.3633 | C_30_H_48_O_3_ | Ursolic acid | Heim et al. 2012 |
| SB1 | [M-H]^-^ | 471.3479 | 427.3579,409.3479 | C_30_H_48_O_4_ | Alpha-hydroxy ursolic acid | Heim et al. 2012 |
| SB1 | [M-H]^-^ | 453.3376 | 409.3776 | C_30_H_46_O_3_ | Tomentosolic acid | Amusan et al. 1996  Heim et al. 2012 |
| SB1 | [M-H]^-^ | 455.3531 | 411.3633 | C_30_H_48_O_3_ | Ursolic acid | Lifongo et al. 2014  Amusan et al. 1996  Heim et al. 2012 |
| SB1 | [M+H]^+^ | 153.0546 | 111.0443 | C_8_H_8_O_3_ | Methyl 4-hydroxybenzoate | Pianaro et al. 2007 |
| SD2 | [M-H]^-^ | 455.3531 | 411.3633 | C_30_H_48_O_3_ | Ursolic acid | Heim et al. 2012 |
| SE1 | [M-H]^-^ | 447.0933 | 285.0402,151.0027 | C_21_H_20_O_11_ | Kaempferol 3-glucoside | Nazif 2007 |
| SE1 | [M+H]^+^ | 449.1078 | 287.0551 | C_21_H_20_O_11_ | Kaempferol 3-glucoside | Nazif 2007 |
| SE3 | [M-H]^-^ | 285.0405 | 151.0025,133.0283 | C_15_H_10_O_6_ | Kaempferol | Lifongo et al. 2014 |
